# A new species of *Lomechusoides* (Coleoptera, Staphylinidae) from *Formica polyctena* anthills in Northeastern Europe

**DOI:** 10.3897/zookeys.1275.182781

**Published:** 2026-03-30

**Authors:** Hjalte Sebastian Kjærby, Sean Birk Bek Craig, Philip Francis Thomsen, Aslak Kappel Hansen

**Affiliations:** 1 Aarhus University, Department of Biology, Ny Munkegade 116, 8000 Aarhus, Denmark Aarhus University, Department of Biology Aarhus Denmark; 2 Aarhus University, Department of Agroecology, Ole Worms Allé 3, 8000 Aarhus C, Denmark Aarhus University, Department of Agroecology Aarhus Denmark; 3 Natural History Museum Denmark, University of Copenhagen, Universitetsparken 15, 2100 Copenhagen E, Denmark Natural History Museum Denmark, University of Copenhagen Copenhagen Denmark

**Keywords:** Ant guests, host specificity, myrmecophiles, social parasitism

## Abstract

*Lomechusoides umbrosus***sp. nov**. (Coleoptera: Staphylinidae: Aleocharinae: Lomechusini) is described, a socially integrated myrmecophile associated with *Formica polyctena* Foerster, 1850. The species is documented from Sweden, Finland, Estonia, Poland, Belarus, and Western Russia (incl. Ural Mountains) based on both newly collected and museum specimens. Field observations show beetles on the surface of host mounds and within nest material, indicating a close association with red wood ant hills. Morphological comparison of the new species and similar *Lomechusoides* species is provided, including an identification key for *Lomechusoides* species of Northern Europe, and an update to the global *L. strumosus* group key. A detailed account of differences between the new species and *L. strumosus* (hosted by *F. sanguinea*), for which it has until now been misidentified as, is made. Mitochondrial COI barcodes are provided to support the morphology and show low but robustly supported genetic divergence (0.7%) between *L. umbrosus***sp. nov**. and *L. strumosus*, which are similar to divergences between other currently recognised species in the genus. Key diagnostic characters include the shape of the pronotum, colour and microsculpture of the head, pronotum and abdomen, dimensions of the antennae, genital structures, as well as the ant host. Our study clarifies long-standing confusion over records of “*L. strumosus*” from nests of the *Formica rufa* group and suggests that host specificity is prevalent within *Lomechusoides*. The holotype is deposited at the Natural History Museum Denmark.

## Introduction

Ant communities are famously ecologically impactful and harvest resources in their environment efficiently. The accumulation of these resources in the ant’s food reserves and within the ants themselves and their waste, are very attractive to other organisms which have evolved to utilise them. This has led to many examples of facultative and obligate myrmecophily across taxa, showcasing a magnificent array of strategies, e.g., from opportunistic predators or detritivores to highly integrated social parasites (Härkönen and Sorvari 2014; Parmentier et al. 2014). The host ants are not welcoming of intruders, driving the evolution of often intricate mechanisms for circumventing the hosts’ defences in myrmecophiles.

Myrmecophiles display varying degrees of host specificity. Species like *Myrmecophilus acervorum* (Panzer, 1799) (Insecta: Orthoptera) and *Platyarthrus hoffmannseggi* Brandt, 1833 (Isopoda: Oniscidea) are known to occur with many ant species of several genera, even across subfamilies (Franc et al. 2015; Parmentier et al. 2017). Other species are strictly host specific, being found only in nests of one or a few closely related ant species. Knowledge on host relations and specificity is unresolved for many myrmecophiles. In addition to lacking records of host relationships for many species, our understanding of host specificity of myrmecophiles is dependent on correct identifications of both myrmecophiles and host ants. Additionally, uncritical associations drawn from incidental occurrences can confound our understanding of host specificity. Taxonomic treatments of some genera such as *Thiasophila* Kraatz, 1856 have also changed our understanding of host specificity within these groups.

In the genus *Thiasophila* Kraatz, 1856., the cryptic species *T. szujeckii* Zagaja & Staniec, 2015 was recently recognised as distinct from *T. angulata* (Erichson, 1837), and the new species appear to show strict host specificity. *Thiasophila szujeckii* is associated solely with *Formica truncorum* Fabricius, 1804, whereas *T. angulata* is found with *F. rufa* Linnaeus, 1761 and *F. polyctena* Foerster, 1850, and *T. lohsei* Zerche, 1987 with *F. pratensis* Retzius, 1783 (Zagaja and Staniec 2015). A similar pattern of host specificity is also seen in the obligately myrmecophilic, *Formica*-associated members of the genus *Oxypoda* Mannerheim, 1830, with *F. rufa/polyctena, F. pratensis*, and *F. exsecta* Nylander, 1846 each hosting a specialised member of the *O. formiceticola* complex (Zerche 1986). Likewise, the genus *Dinarda* Leach, 1819 has long been known to show host specificity within *Formica*. *Dinarda dentata* (Gravenhorst, 1806) is hosted by *F. sanguinea* Latreille, 1798, *D. hagensii* Wasmann, 1889 by *F. exsecta*, *D. maerkellii* Kiesenwetter, 1843 by the *F. rufa* group, and *D. pygmaea* Wasmann, 1894 by *F. rufibarbis* Fabricius, 1793 and *F. cunicularia* Latreille, 1798 (Wasmann 1894).

The obligate myrmecophile genus *Lomechusoides* Tottenham, 1939 comprises incredibly sophisticated kleptoparasites, being socially integrated into the nests of their host ants (Wasmann 1915; Hölldobler et al. 2018). Their behaviour has been closely studied in captivity for more than a century, and some early researchers were convinced that *Lomechusoides* and the related genus *Lomechusa* Gravenhorst, 1806 both exhibited relatively strict host specificity for different *Formica* species (Wasmann 1915). However, information regarding their host relationships have since been increasingly muddied. The most well-studied *Lomechusoides* species, *L. strumosus* (Fabricius, 1792), has for a long time been perceived as an obligate parasite of *F. sanguinea*, only occurring with other ant species in very rare, accidental cases (Wasmann 1915). Contrastingly, modern papers list a multitude of ant species as hosts of *L. strumosus*, often without highlighting the importance of *F. sanguinea* in any way. In addition to *F. sanguinea*, this list includes much of the *F. rufa* group, *F. fusca* Linnaeus, 1758, *F. rufibarbis* Fabricius, 1793, and even species of *Myrmica* Latreille, 1804 (Hlaváč et al. 2011; Harrison and Albena 2014; Päivinen et al. 2002; Jászay et al. 2023). Such promiscuous and seemingly opportunistic host choice seems unlikely for a highly integrated myrmecophile, especially in light of the host-specific *Thiasophila, Oxypoda*, and *Dinarda* species, which are not socially integrated. We hypothesise that the long list of hosts for *L. strumosus* might originate from misinterpretations of accidental occurrences, and/or erroneous identifications of both beetles and ants in the past.

Following this suspicion of potentially misrepresented host infidelity, HSK collected multiple *Lomechusoides* specimens from mounds of *Formica polyctena* at Hammarskog near Uppsala, Sweden. In this region, morphologically and ecologically suspicious *Lomechusoides* specimens had been repeatedly reported on Artportalen.se as “*L. strumosus*”. These specimens and conspecifics identified from museum collections do not conform to any described species and we therefore provide a description of this enigmatic and overlooked new species. Additionally, we provide an update to the most recent key to the *Lomechusoides* species in the *L. strumosus* group, as well as a key limited to the *Lomechusoides* species of Northern Europe. We also provide a detailed morphological comparison of the new species and *L. strumosus*, for which it has been most commonly misidentified, facilitating correct identification of the new species.

## Materials and methods

The finding of *Lomechusoides* specimens on the surface of *Formica polyctena* anthills in Hammarskog, Uppsala, Sweden prompted this study, as they could not be appropriately assigned any current species, despite the recent and very extensive revision by Jászay et al. (2023). Additional comparative material was gathered from the following depositories:

**NHMA** Natural History Museum Aarhus, Aarhus, Denmark (Thomas Simonsen);

**DBET** Collection of the Department of Biodiversity and Evolutionary Taxonomy, University of Wroclaw, Poland (Rafał Ruta and Lech Borowiec);

**NHMD** Natural History Museum of Denmark, Copenhagen, Denmark (Alexey Solodovnikov);

**NHMW** Naturhistorisches Museum, Wien, Austria (Harald Schillhammer);

**MZLU** Lund University Biological Museum – Zoological collections, Lund, Sweden (Christoffer Fägerström);

**SDEI** Senckenberg Deutsches Entomologisches Institut, Müncheberg, Germany (Vinicius Ferreira);

**SMNS** Staatliches Museum für Naturkunde, Stuttgart, Germany (Arnaud Faille);

**TAMZ** Estonian Museum of Natural History – Zoological collections, Tallinn, Estonia (Uno Roosileht);

**UiT** Norges Arktiske Universitetsmuseum, Tromsø, Norway (Jostein Kjærandsen);

**ZMUH** Universität von Hamburg, Museum der Natur Hamburg, Hamburg, Germany (Dagmara Żyła);

**ZMUO** Zoological Museum of Oulu University, Oulu, Finland (Mikko Vallinmäki);

**cHans** private collection of Oddvar Hanssen, Norway;

**cKjær** private collection of Hjalte Kjærby, Aarhus, Denmark;

**cMart** private collection of Ole Martin, Denmark;

**cSem** private collection of Oleg Semionenkov, Smolensk, Russia;

**cWann** private collection of Hans-Erik Wanntorp, Sweden;

**cØde** private collection of Frode Ødegaard, Norway.

Details about all studied material is available in Suppl. material 2: table SS1.

It should be noted that Fabricius’ original type material of *Lomechusoides strumosus* from Saxony is currently lost. We follow the description of *L. strumosus* as defined by Jászay et al. (2023), although a few of the specimens assigned by these authors to *L. strumosus* have been determined by us to be *L. umbrosus* sp. nov., specifically the Vittsjö series. Based on host ant and fig. 48 in Jászay and Hlaváč (2013) we deem it likely that the Bardejov series also represent the new species, but we have not examined this material personally.

To allow accurate comparison with recent work on the *L. strumosus* species group, all morphological characters used in the species descriptions in Jászay et al. (2023) have been described for the new species, and the description has been worded and structured similarly. Nomenclature follows StaphBase on Catalogue of Life (Newton 2026)

Measurements were done on Enersight v. 1.2.0.56 on images taken with Leica Ivesta 3 (Integrated camera), and are all in mm. The following abbreviations are used:

**TL** total length of whole body;

**FL** forebody length;

**AL** length of antenna;

**HW** width of head measured across eyes;

**HL** length of the head capsule;

**PW** maximum width of the pronotum;

**PL** length of the pronotum measured along median line;

**EW** maximum width of elytra;

**EL** length of elytra measured along the suture;

**FTL** length of the fore tibia;

**MTL** length of the mid tibia measured;

**HTL** length of the hind tibia.

### Photography and figures

Habitus images were made using a Canon EOS 7R camera fitted with a Canon MP-E65 f2.8 1–5× macro lens attached to a Cognysis StackShot Macro Rail to produce stacks. They consisted of stacks of 30 images combined using Zerene Stacker v. 1.04 (Zerene Systems, Richland, WA, USA) and stacked using the Pmax function. Images of genitalia were taken with Leica Ivesta 3 (Integrated camera) with Enersight v. 1.2.0.56 software. The final images were edited in Lightroom v. 8.5.1 (Adobe Inc., San Jose, CA, USA) to adjust colour and exposure and Photoshop v. 26.11.0 (Adobe Inc., San Jose, CA, USA) to remove dust and other impurities. Final figures including line drawing were made and assembled in Illustrator v. 29.8.2 (Adobe Inc., San Jose, CA, USA).

### DNA barcoding and genetic species delimitation

DNA barcoding was performed through extractions using the HotShot method (Truett et al. 2000), with an initial lysis of tissue using heated alkaline buffer solutions (16 min at 65 °C and 4 min at 98 °C), followed by neutralisation to pH 7. PCR was performed using a tagged primer approach targeting a 418 bp fragment of cytochrome oxidase I (COI) using the primers BF3 and BR2 (Elbrecht and Leese 2017; Elbrecht et al. 2019). We followed (Srivathsan et al. 2021, 2024) adding a 9-bp tag to the 5’ end of the primers, allowing for multiplexing of several PCR products from multiple samples on a single flowcell for sequencing. All PCR were set up with 2 μl HOT FIREPol Blend Master Mix Ready to Load, 1 μl F primer, 1 μl R primer,4 μl (HotShot) DNA template dependent on extraction method, and lastly ddH2O to make up a total of 15 μl. We used the following PCR conditions: (15 min 95 °C – 35x (30 s 94 °C – 1.5 min 50 °C – 1.5 min 72 °C) – 10 min 72 °C). PCR products were pooled, cleaned using AMPure XP beads, and adapters were ligated before sequencing on a Nanopore MinION MK1C using a Flongle or MinION flow cell (R.10.4.1). Basecalling was conducted through Dorado (0.9.1) and raw fastq files were imported to OntBarcoder2.3 for consensus barcode calling (Srivathsan et al. 2024), these were further verified by mapping on the tree and blasting to mkCOInr database (v3, May 2024) to filter clear obvious non- target sequences (Meglécz 2023). A final alignment was assembled by adding novel data passing above quality controls together with sequences harvested from BOLD and GenBank of *Lomechusoides*. Details about all barcoded material is given in the Suppl. material 3: table SS2.

All novel and publicly available COI sequences from BOLD and GenBank for *Lomechusoides* and *Lomechusa* species were compiled with a single sequence of *Drusilla canaliculata* (Fabricius, 1787) used as an outgroup. The entire dataset was aligned using the MAFFT Multiple Alignment (v. 1.5.0) plugin in Geneious Prime (Katoh et al. 2002). The aligned dataset is available from Suppl. material 1.

Two separate phylogenetic analyses, Maximum Likelihood (ML) and Bayesian Inference (BI) were performed. The ML analysis was conducted using the IQ-TREE 2 with default settings, except partitioning by codon position, and support was evaluated by 1000 iterations of Ultrafast Bootstrap (UFP) and Shimodaira–Hasegawalike approximate likelihood ratio test (SH-aLRT), respectively. Model search for the BI dataset was initially run in ModeFinder of IQ-TREE 2, with partitioning by codon position and restricted to models available in MrBayes. Using the best fit model, the BI analysis was performed using MrBayes (v. 3.2.7) (Ronquist et al. 2012) with default settings with support as a posterior probability (PP). Support values are reported in the following format (PP/UFP/SH-aLRT), Lastly a TCS haplotype network was produced using Popart 1.7 (Leigh and Bryant 2015). The final figure was assembled and colourised in Illustrator v. 29.8.2.

## Results

### DNA barcoding and species delimitation

Of a total of 17 specimens of *Lomechusa* and *Lomechusoides*, 12 were successfully sequenced, yielding high-quality COI barcodes of 418 bp length. These were combined with 14 sequences from BOLD and GenBank to produce an alignment containing 26 DNA barcodes.

**Figure 1.**
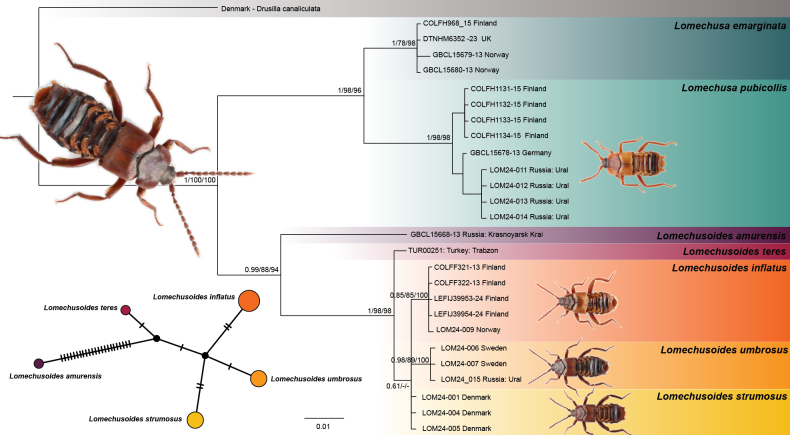
A TCS haplotype network and consensus phylogenetic tree based on Bayesian Inference (BI) and Maximum Likelihood (ML) analyses of the COI barcode, with support values. Support values at each node are shown in the following order: PP (BI), UFB (ML), SH-aLRT (ML). The size of the circles in the haplotype network is proportional to the number of specimens in the clusters; vertical bars between the clusters represent nucleotide differences.

Both Maximum Likelihood (ML) and Bayesian Inference (BI) analyses produced congruent topologies, supporting the reciprocal monophyly of *Lomechusoides* (PP=0.99/ UFP=88/ SH-aLRT=94) and *Lomechusa* (PP=1/UFP=98/SH-aLRT=96) (Fig. 1) as sister taxa. Within the genus *Lomechusoides*, 5 clades were recovered corresponding to the five sampled species *L. amurensis* (Wasmann, 1897), *L. teres* (Eppelsheim, 1884), *L. inflatus* Zetterstedt, 1828, *L. strumosus*, and *L. umbrosus* sp. nov. Four of these were supported by the analysis, *L. amurensis* (PP=0.99/ UFP=88/ SH-aLRT=94), *L. teres* (PP=1/ UFP=98/ SH-aLRT=98), *L. inflatus* (PP=0.85/ UFP=85/ SH-aLRT=100), and *L. umbrosus* sp. nov. (PP=0.98/ UFP=89/ SH-aLRT=), with only *L. strumosus* (PP=0.61/ UFP=na/ SH-aLRT=na) not being properly recovered in any analysis. Likely the low genetic distance between all these resulted in difficulties with recovering all species in a phylogenetic context.

The average pairwise tree distances between the species within *Lomechusoides* were generally low, except for *L. amurensis* that showed 7.0% distance to all other sampled members. Within the four remaining sampled members *L. teres*, *L. inflatus*, *L. strumosus*, and *L. umbrosus* sp. nov. the pairwise distance was between 0.7–1.4%. The lowest distances (0.7%) were between *L. strumosus* and all other sampled members *L. teres*, *L. inflatus*, and *L. umbrosus* sp. nov. The TCS haplotype network revealed small but clear separation between all sampled *Lomechusoides* species (Fig. 1). Only *L. amurensis* was very different from other species with 20+ bp differences across the 418 bp sequence studied, other species diverged 3 to 4 bp across the same fragment.

Together, these results, although small, support the existence of a genetically distinct previously unknown lineage with similar divergence as other already recognised species within the genus.

### Taxonomy

**Staphylinidae Latreille, 1802**

**Aleocharinae Fleming, 1821**

**Lomechusini Fleming, 1821**

***Lomechusoides* Tottenham, 1939**

#### Lomechusoides umbrosus sp. nov.

Taxon classificationAnimaliaColeopteraStaphylinidae

E02A9F0D-C676-54DE-84EE-FF46F64562B3

https://zoobank.org/DBA3B3D1-5BDC-4ACC-8107-81244D93B62B

**Figure 2.**
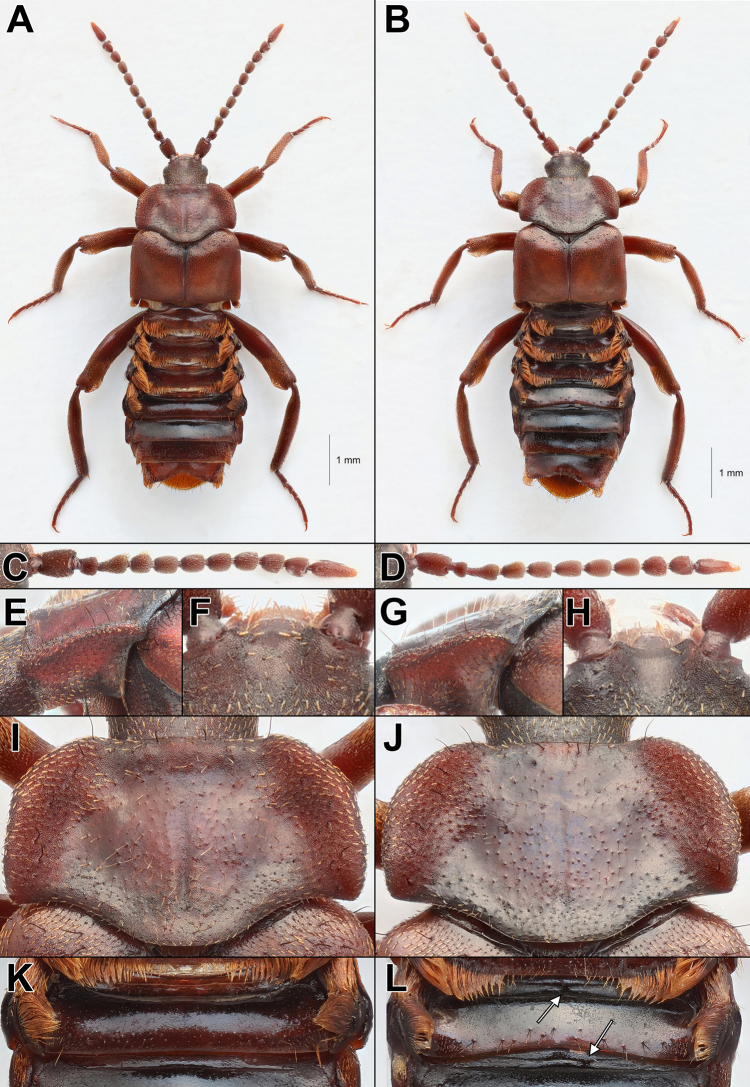
Morphological comparison of **A**. *Lomechusoides strumosus* (Male. w. *Formica sanguinea*, Feldborg Nørreskov, Jutland, Denmark); **B**. *L. umbrosus* sp. nov. (Male holotype. w. *F. polyctena*, Hammarskog, Uppsala, Sweden). The antennomeres, especially the 3^rd^ segment, of *L. strumosus* (**C**) are thicker and shorter than in *L. umbrosus* sp. nov. (**D**). From lateral view, the pronotal lateral margins have a blunt edge in *L. strumosus* (**E**), while they have a short sharp edge posteriorly in *L. umbrosus* sp. nov. (female paratype) (**G**). The frontal median impression of the brown head is dull with clear, even microsculpture in *L. strumosus* (**F**), while the head is black and the frontal median impression is shiny with weak, uneven microsculpture in *L. umbrosus* sp. nov. (**H**). The pronotal disc is dull with even light microsculpture, pronotal margins subparallel posteriorly, suddenly narrowing frontally, and with widest point at or immediately anterior thereof in *L. strumosus* (**I**). The pronotal disc is very glossy with almost no microsculpture, lateral margins smoothly curved in their entire length, with widest point at ¼–⅓ of the marginal length from the anterior corners in *L. umbrosus* sp. nov. (**J**). Transverse carinae of the anterior part of tergite II–V unconnected in their entire length in *L. strumosus* (**K**), while they are connected in the centre of the tergite by a broad septum (marked with arrows), occasionally two or more broad septa, in *L. umbrosus* sp. nov. (**L**).

Figs 2A, 2C, 2E, 2F, 2I, 2K, 7A

##### Type locality.

Hammarskog, Uppsala, Uppland, Sweden. 59.774586, 17.578827.

##### Material examined.

***Holotype*: Sweden** • 1♂; Hammarskog, Uppland; 59.774586, 17.578827; 20.IX.2023; leg. H. Kjærby; w. *Formica polyctena*; NHMD. ***Paratypes*: Belarus** • 4♂♂, 1♀; Vitebsk reg., Polotsk distr., 3 km SW of Novopolotsk, near the lake Luechovo, 55.5168, 28.6318, 152 m, in the nest of *Formica polyctena*, fir-wood, sorrel, 30.V.2013, leg. E. Pliskevich; cSem • 1♂; Vitebsk reg., Polotsk distr., 3 km SW of Novopolotsk, near the lake Luechovo; 55.5167, 28.6325; 152 m; 10.VI.2015; leg. E. Pliskevich; in the nest of *Formica polyctena*, fir-wood, sorrel; cSem. **Russia** • 2♀♀; Smolensk distr., near Vysokoye; 54.73, 32.21; 23.V.–11.VI.2022; leg. O. Semionenkov; window trap near dead spruce and nest of *Formica polyctena*, coniferous-broad-leaved forest; cSem • 1♂, 3♀♀; Svedlovsk Reg., Visimskij Nature Res.; 57.3734, 59.7734; 570 m; 18.VI.2019; leg. A. Solodovnikov, A.K.Hansen, A.Tokareva; spruce dominated forest, in *Formica* nests; NHMD. **Sweden** • 1♀; Hacksta, Uppland; 59.55, 17.38; 30.IV.2022; leg. H.-E. Wanntorp; w. *Formica polyctena*; cWann • 1♂; Hållvik, Uppland; 59.93, 18.41; 12.V.2025; leg. H.-E. Wanntorp; cWann • 3♂♂, 4♀♀; Hammarskog, Uppland; 59.77, 17.57; 20.IX.2023; leg. H. Kjærby; w. *Formica polyctena*; cKjær • 6♂♂, 3♀♀; Skåne, Vittsjö; 56.34, 13.66; *Formica rufa* nest; 24.V.1980; leg. A. Carlson; MZLU.

##### Additional material.

**Estonia** • 1♂; Välgi; 58.55, 26.92; 18.V.–7.VII.2024; forest edge window trap on dead spruce; TAMZ. **Finland** • 1♀; Karelia australis, Joutseno, Kuurmanpohja; 61.073, 28.729; 71 m; 8.VI.2015–11.VII.2015; leg. Eero Helve; window trap, mushroomed log pile (aspen, spruce, birch); ZMUO. **Poland** • 1♀; Bialowieza, Czerlonka; 52.69, 23.72; 19.VI.1991; leg. L. Borowiec; DBET.

##### Differential diagnosis.

The new species can be separated from all other members of the *Lomechusoides strumosus* group sensu Jászay et al. (2023) by the following combination of characters: 1) widest point of the pronotum positioned at ~ ¼–⅓ of the length of the pronotal lateral margin from the posterior corners, making the posterior corners distinctly obtuse. 2) pronotal lateral margins smoothly rounded in their entire length 3) a visible sharp margin on the posterior curved part of the pronotal margins (best viewed laterally and slightly from behind) (Fig. 2G). 4) pronotal disc glossy with very limited, uneven microsculpture, but with very well-defined tubercles. 5) head, sternites and tergites predominantly black. 6) clypeus and frontal median impression of the head shiny with limited, uneven microsculpture. 7) two transverse carinae on the anterior part of visible tergites II to V connected in the centre of the tergite by a broad septum, occasionally two or more broad septa, rarely absent.

See Table 2 for a comprehensive list of identified traits that separate *L. umbrosus* sp. nov. from its close congener *L. strumosus*.

**Table 1.** Genetic distances between sampled species given in (upper right) number of nucleotide differences across the studied 418 bp fragment of the DNA barcode and as (lower left) average pairwise tree distance in percentage.

	*L. umbrosus*	*L. strumosus*	*L. inflatus*	*L. teres*	*L. amurensis*
*L. umbrosus*		3	3	3	25
*L. strumosus*	0.7		4	4	26
*L. inflatus*	1.4	0.7		4	26
*L. teres*	0.9	0.7	1.4		24
*L. amurensis*	8.0	7.7	8.5	7.0	

**Table 2.** Detailed morphological comparison between *Lomechusoides strumosus* and *L. umbrosus* sp. nov.

Species	*Lomechusoides strumosus*	*Lomechusoides umbrosus* sp. nov.
Host ant	**Formica (Raptiformica) sanguinea**	**Formica (Formica) polyctena**, and possibly other *F. rufa* group species (*rufa*, *aquilonia*, *lugubris*, *pratensis*)
Pronotum shape (dorsal view)	The widest point of the pronotum is at the posterior corners (Fig. 7A) or immediately anterior thereof (Fig. 2I). The posterior ~ ⅔ of the lateral margins are subparallel, then abruptly narrowing in anterior third, creating an angled bend at ~⅓ of the length of the lateral margins from anterior corners.	The widest point of the pronotum at ~¼–⅓ of the lateral marginal length from the posterior corners (Figs 2J, 7A). The lateral margins are smoothly rounded as the pronotum narrows anteriorly. Behind the widest point, the pronotum narrows posteriad. Generally, the lateral margins are convex and smoothly rounded in their entire length, giving them a semicircular shape.
	Hind corners backward-protruding, often making them slightly acute (with some slight variations depending on the degree of posterior narrowing).	Hind corners not backward-protruding, making them slightly obtuse.
Pronotum shape (lateral view)	In lateral view, pronotal lateral margins are thin with blunt edge in curved part before posterior corners (Fig. 2E).	In lateral view, pronotal lateral margins are very thin with visible sharp edge in curved part before posterior corners (Fig. 2G). Slight individual variation occurs, with the length of sharp edge-bearing margin being longer and more easily recognised in some specimens than others.
	*Few specimens from Austria have a long, sharp edge of the pronotal margins. These specimens deserve further attention.	
Pronotum colour and microsculpture	Pronotal lateral margins have the same reddish-brown colour as the pronotal disc.	Pronotal lateral margins are paler reddish-brown in colour than the darker deep bordeaux pronotal disc.
	Pronotal disc with light microsculpture all over, quite dull (Fig. 2I).	Pronotal disc with little to no microsculpture, very glossy (Fig. 2J).
Antennae	Antennomeres robust, and antennae relatively short. Third segment short, at most 1.5 × longer than wide, often slightly shorter. Remaining antennomeres thick, resembling pearls on a string (Fig. 2C).	Antennomeres relatively gracile, and antennae longer. Third segment long, ~1.8–2 × longer than wide. Remaining antennomeres relatively slender, somewhat intermediate between *L. strumosus* and *L. wellenii* (Fig. 2D).
Head	Head brown, eyes flat or slightly protuberant, temples straight or weakly narrowed behind eyes.	Head black, eyes slightly to strongly protuberant, temples weakly to strongly narrowed behind eyes.
	U-shaped frontal median impression densely and evenly microsculptured, almost as strongly as the rest of the head, dull (Fig. 2H).	V-shaped frontal median impression weakly and unevenly microsculptured, much weaker than the rest of the head, shiny (Fig. 2F).
	Clypeus densely microsculptured, dull.	Clypeus weakly microsculptured, shiny.
Abdomen colour	Tergites and sternites (Fig. 7A) mostly reddish-brown, except visible tergite V which is mostly blackish-brown with a thin reddish-brown transverse stripe at posterior margin, and tergite VI which is blackish-brown in anterior half, and reddish-brown in posterior half.	Tergites and sternites (Fig. 7B) mostly black, each with a thin reddish-brown stripe along posterior margins.
Tergite structure	Two transverse carinae on the anterior part of visible tergites II–V unconnected in their entire length (Fig. 2K).	Two transverse carinae on the anterior part of visible tergites II–V connected in the centre of the tergite by a broad septum (Fig. 2L), occasionally 2 or more broad septa. In a few specimens from Vitebsk and Smolensk the carinae are unconnected.
Sternite structure	Sternite VI densely punctate, each puncture with a short, golden hair. Max distance between punctures is ~ 2 × diameter of punctures.	Sternite VI more sparsely punctate, each puncture with a short, golden hair. Max distance between punctures is ~ 4 × diameter of punctures.
Spermatheca	Basal part of spermatheca distinctly longer than apical part; Basal part 1.21–1.53 × length of apical part (Fig. 4E).	Apical and basal parts of spermatheca approximately equal in length; Basal part 0.88–1.16 × length of apical part (Fig. 4F).
	Spermatheca often paler amber-coloured.	Spermatheca often darker brown.
Aedeagus (median lobe)	In dorsoventral view, the apex is relatively broad, roughly shaped like an equilateral or isosceles triangle (Fig. 4A).	In dorsoventral view, the apex is thin, roughly shaped like an acute triangle (Fig. 4B).
	In lateral view subapically with slight concave expansion, but without distinct expansion and clear notch (Fig. 4C).	In lateral view with a distinct subapical expansion followed by a notch (Fig. 4D).
	In lateral view, the entire dorsal margin of the chitinised part of the median lobe is sigmoid (S-curved) (Fig. 4C).	In lateral view, the entire dorsal margin of the chitinised part of the median lobe is concave (Fig. 4D).

##### Description.

**Female**. Measurements (mm): TL 6.81; FL 3.07; AL 3.34; HW 0.91; HL 0.94; PW 1.99; PL 1.20; EW 2.24; EL 0.96; FTL 1.30; MTL 1.66; HTL 1.95. Ratios: HW/HL 0.96; PW/PL 1.66; EW/EL 2.33.

**Male**. Measurements (mm). TL 6.46; FL 2.88; AL 2.90; HW 0.84; HL 1.08; PW 1.72; PL 1.04; EW 2.07; EL 0.94; FTL 1.25; MTL 1.47; HTL 1.75. Ratios: HW/HL 0.78; PW/PL 1.65; EW/EL 2.2.

***Colouration*** dark. Legs and antennae dark reddish-brown. Head entirely black. Pronotal disc deep bordeaux reddish-brown, with a semicircular blackish pattern along the posterior margin; the pronotal lateral margins are paler reddish-brown than the pronotal disc. Elytra almost entirely bright reddish-brown, blackish-brown along anterior margins. Thorax with sterna black. Tergites and sternites almost entirely black, each with a thin reddish-brown transverse stripe along the posterior margins.

***Head*** rectangular, with deep V-shaped frontal median impression very weakly microsculptured, shiny, very sparsely and finely punctate (Fig. 2H). A central spot at the hind part of the head without microsculpture. Clypeus finely and unevenly microsculptured, shiny. The rest of the head is evenly microsculptured and finely punctate, dull. Eyes slightly to strongly protuberant. Temples overall concave, usually being strongly narrowed immediately behind the eyes, subparallel posteriad.

**Figure 3.**
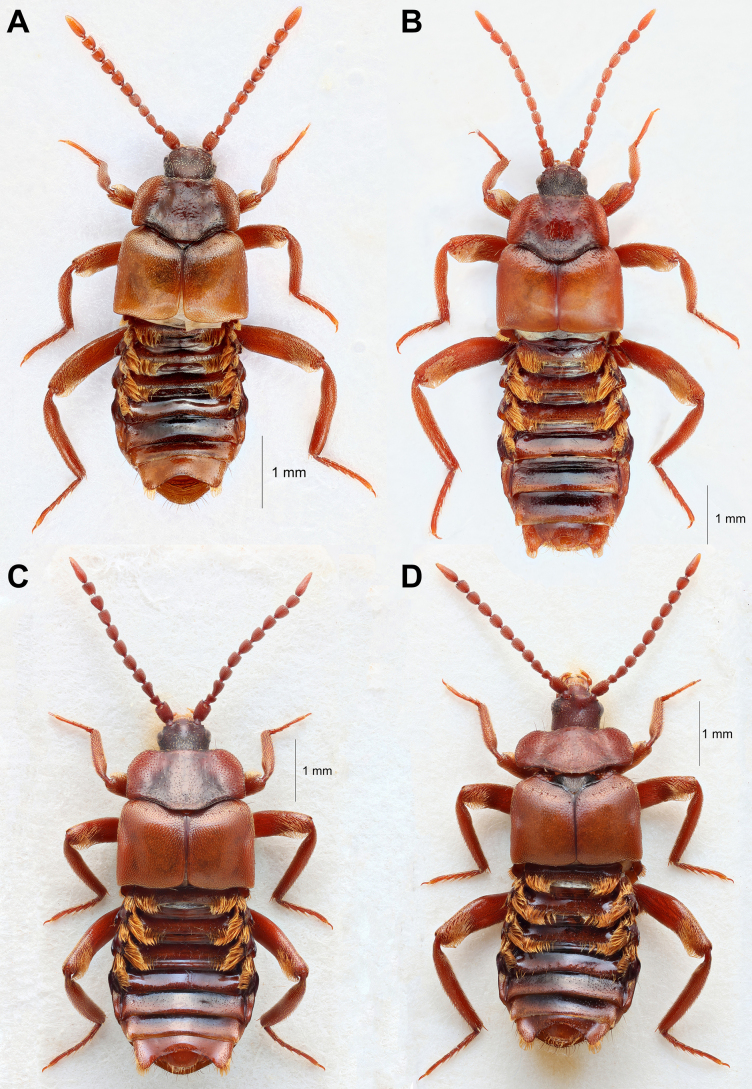
The two other Nordic *Lomechusoides* species *L. inflatus* (**A**) and *L. wellenii* (**B**), and the morphologically most similar species to *L. umbrosus* sp. nov., *L. primoricus* (**C**), and *L. zerchei* (**D**) from East Siberia.

***Antennae*** (Fig. 2D) relatively gracile and long (Fig. 3B). Male with dense tufts of golden pubescence on antennal segments III–IV.

**Male**. Scape 1.52 × as long as wide, and 1.8 × as long as pedicel; pedicel 1.31 × as long as wide; antennomere III 1.89 × as long as wide; antennomere IV 1.4 × as long as wide; antennomeres V–VIII all ~ 1.5 × as long as wide; terminal antennomere slender and acute, 3.25 × as long as wide, 1.36 × as long as scape.

**Female**. Scape 1.6 × as long as wide, and 1.68 × as long as pedicel; pedicel 1.26 × as long as wide; antennomere III 1.86 × as long as wide; antennomere IV 1.41 × as long as wide; antennomeres V–VIII all ~ 1.4–1.6 × as long as wide; terminal antennomere slender and acute, 2.9 × as long as wide, 1.37 × as long as scape.

***Pronotum*** (Fig. 2J) with concave anterior margin; anterior corners obtuse; with visible microsculpture along anterior and posterior margins, and in depressions. Pronotal lateral margins are smoothly rounded, semicircular convex and with strong microsculpture. The widest point of the pronotum ~ ¼–⅓ of the lateral marginal length from the posterior corners. As the pronotum narrows from the widest point towards the anterior corners, the lateral margins are evenly rounded without angular bends. Behind the widest point, the pronotum narrows slightly posteriorly, making the posterior corners distinctly obtuse. This results in the pronotal lateral margins being smoothly rounded in their entire length.

In lateral view, the pronotal lateral margins are thick in the anterior half, then abruptly thinning in the posterior half. At posterior corners the margin is strongly curved and very thin with a visible sharp edge (Fig. 2G). The length of sharp edge-bearing margin varies but is usually rather short and constrained to the curved part at posterior corners.

The pronotal lateral margins are separated from the pronotal disc by shallow longitudinal depressions with the lateral area with visible microsculpture. Pronotal disc (Fig. 2J) with either very limited, unevenly distributed fine microsculpture, or no microsculpture at all, giving the central part of the pronotum a very glossy appearance. Pronotal disc with well-defined tubercles and median line. Pronotal tubercles are very noticeable due to the lack of surrounding microsculpture. Pronotal tubercles bearing long, thin erect golden pubescence. Lateral margins of pronotum with 5–7 black macrosetae, anterior margin with 1–4 black macrosetae.

***Metaventral process*** microsculptured and sparsely punctate with concave anterior and lateral margins, possessing lateral lines that converge anteriorly. Anterior part with no/very weakly defined transverse carina, posterior part of median line lacking lentil-like depression. Metaventral disc microsculptured and sparsely punctate with weakly defined longitudinal shallow impressions on either side of the posterior part of the median line.

***Elytra*** finely and evenly punctate and microsculptured. Each puncture bears a short golden hair. Anterior part bearing eight or nine black macrosetae, humeral part with two black macrosetae.

***Abdomen*** with visible tergite I densely punctate and evenly microsculptured; II and III densely punctate along posterior margin, otherwise finely and sparsely punctate, finely, and unevenly microsculptured. Visible tergites IV and VII finely and sparsely punctate and finely and unevenly microsculptured; visible tergites V–VI equally finely and sparsely punctate in median part, lateral parts bearing slightly larger and denser punctures and more visible microsculpture. Visible tergites I–IV with dense golden trichomes laterally, visible tergites I–III additionally with shorter, golden trichomes along posterior margin, thinning towards the centre. Visible tergites V–VII without trichomes. Anterior part of visible tergites II–V bearing two transverse carinae. The two carinae are joined near the centre of the tergite by a septum (Fig. 2L). Occasionally, two or more connecting septa may be present, and rarely they may be absent. Area around the two transverse carinae with wavy microsculpture. Posterior parts of visible tergites I–III, especially II, with numerous black macrosetae. Posterior margins of visible tergites IV–VII with fewer, smaller dark setae.

Sternites I–IV finely microsculptured and sparsely punctate, rather glossy. Sternite V and especially VI more strongly microsculptured and moderately punctate. Max distance between punctures on sternite VI is ~ 4 × the diameter of punctures. Sternites I–III with dense, flat-laying, golden trichomes laterally in the anterior part. All sternites with numerous black macrosetae.

##### Male genitalia.

**Figure 4.**
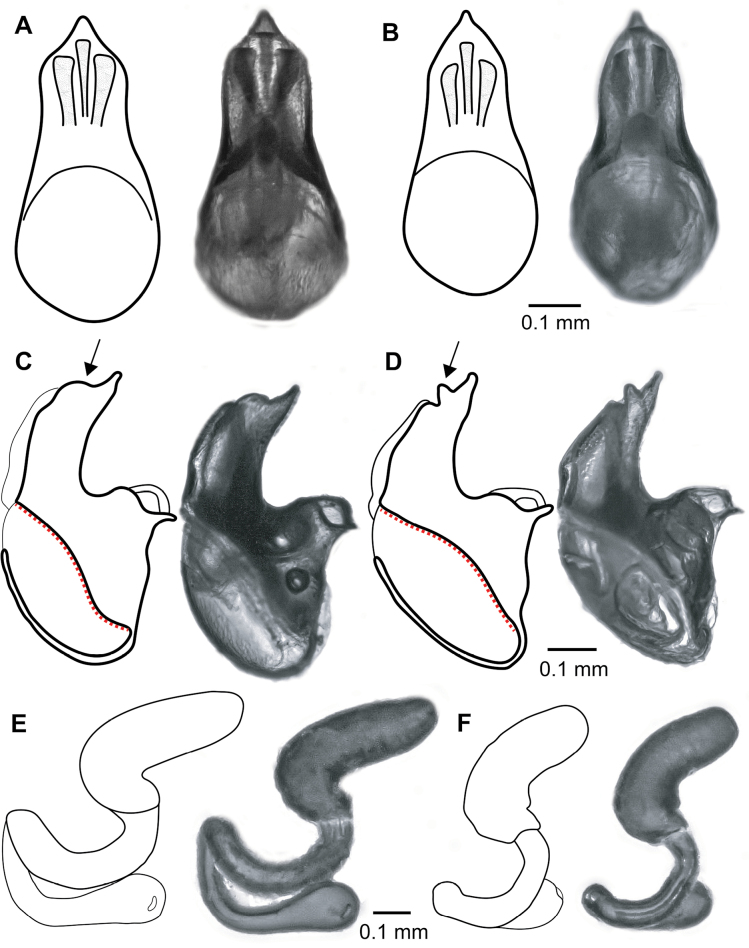
Genitals of *L. strumosus* from Jutland: Dorsal (**A**) and lateral (**C**) view of aedeagus, lateral view of spermatheca (**E**) and *L. umbrosus* sp. nov. from Uppsala: Dorsal (**B**) and lateral (**D**) view of aedeagus, with arrow indicating subapical expansion. Lateral view of spermatheca (**F**).

Aedeagus (Fig. 4B, D) with robust median lobe, apical lobe shorter than basal bulb. In lateral view, apex is thin, with a subapical expansion followed by a notch (Fig. 4D). In lateral view, the entire dorsal margin of the chitinised part of the median lobe is concave. In dorsoventral view, apex is relatively thin, roughly shaped like an acute triangle, sides of median lobe softly concave. In ventral view, lateral sides of apical lobe narrow apically along entire length, most strongly narrowing in basal part. Apex of parameres with a short, backwards-curving tooth.

##### Female genitalia.

Spermatheca (Fig. 4F) with basal and apical parts approximately equal in length; basal part 0.88–1.16 × the length of apical part; tip of apical part rounded. The entire spermatheca is uniformly dark brown.

##### Etymology.

The species epithet *umbrosus*, meaning shadowy in Latin (nominative masculine), refers both to the generally very dark colouration, and its habitat being nests of *Formica polyctena*, who are often found in relatively shaded forest environments.

##### Distribution.

**Figure 5.**
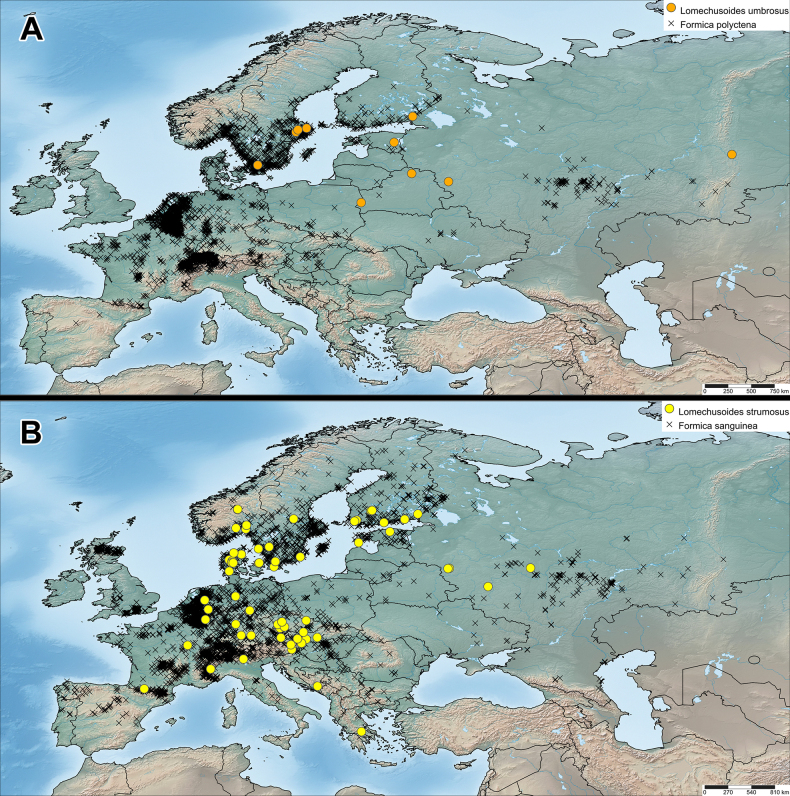
Distribution of **A**. *Lomechusoides umbrosus* sp. nov. (examined material) and *Formica polyctena* (GBIF); **B**. *Lomechusoides strumosus* (examined material) and *F. sanguinea* (GBIF).

*Lomechusoides umbrosus* sp. nov. is known from a few sites in Sweden, Finland, Estonia, Poland, Belarus, and Northwestern Russia (Fig. 5A). In Sweden it is found in the region of Uppland on several sites between Stockholm and Uppsala (Hammarskog, Hacksta, and others), as well as Vittsjö in Scania in southernmost Sweden. In Finland it has been collected on a site in South Karelia. In Estonia it has been found near Tartu. In Poland it is known from Bialowieza Primeval Forest. In Belarus it has been collected near Vitebsk (Novopolotsk), and nearby in Russia it is known from Smolensk (Vysokoye). The second known Russian site is in the Urals (Visimskij Nature Reserve) and marks the easternmost known site for the species.

##### Bionomics.

**Figure 6.**
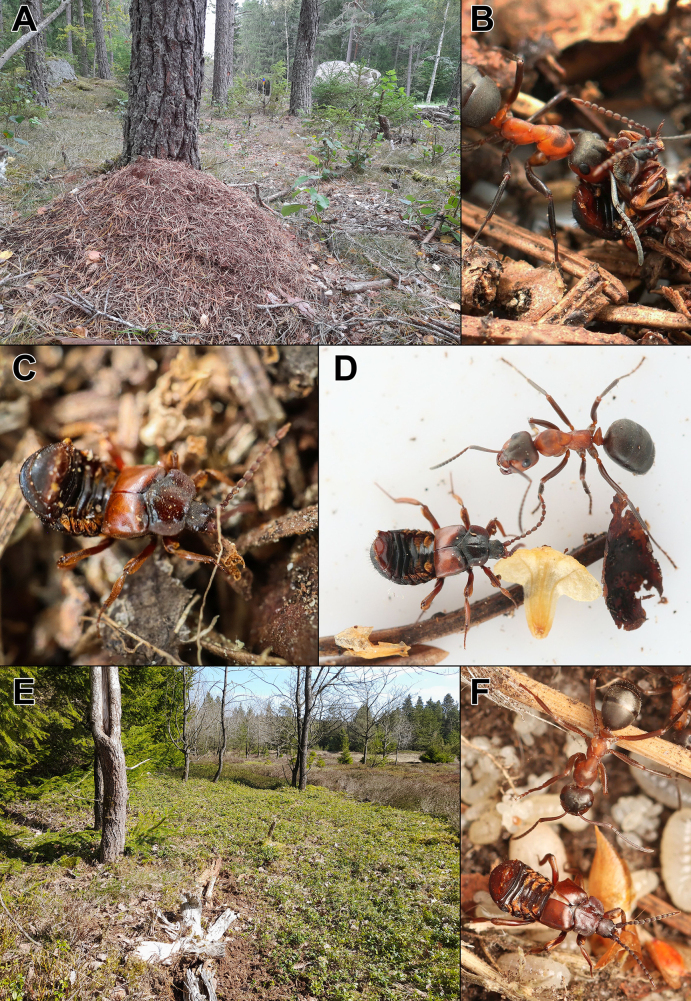
Ecological difference between *Lomechusoides umbrosus* sp. nov. and *L. strumosus*. **A**. *Formica polyctena* nest in a pine forest in Hammarskog, Uppsala, Sweden, where the male holotype of *L. umbrosus* sp. nov. was collected; **B, D**. *Lomechusoides umbrosus* sp. nov. females with host *F. polyctena* workers at the type locality; **C**. *Lomechusoides umbrosus* sp. nov. male sifted from a *F. polyctena* nest in the Ural Mountains; **E**. *Formica sanguinea* nest on heathland at the edge of a spruce plantation in Tingskov, North Jutland, Denmark heavily infested with *L. strumosus*; **F**. *Lomechusoides strumosus* female in a nest of *F. sanguinea* in Kompedal Plantage, Central Jutland, Denmark.

*Lomechusoides umbrosus* sp. nov. is only known from the mounds of *Formica polyctena* (Fig. 6A–D). On several occasions during both spring and autumn, individuals of *L. umbrosus* sp. nov. have been collected on the surface of the *F. polyctena* host anthills, sometimes whilst being carried by workers (HSK pers. obs.; Artportalen.se). On other occasions, *L. umbrosus* sp. nov. has been found by sifting nest material of *F. polyctena* (AKH pers. obs.; Artportalen.se).

Due to its association with *F. polyctena*, *L. umbrosus* sp. nov. is most commonly found in coniferous or mixed forests, often in rather shaded environments (Fig. 6A). It is not strictly associated with this habitat, however. At Hacksta (Vallentuna, Uppland, Sweden), it has been recorded in a *F. polyctena* nest located on a forest-adjacent open pasture (Artportalen.se).

The myrmecophile behaviour of *L. umbrosus* sp. nov. has never been studied. However, it is thought to be similar to the closely related *L. strumosus*, that has been subject to many behavioural studies since the mid 1800’s. *Lomechusoides strumosus* infiltrates host *F. sanguinea* nests through an elaborate adoption process using the pheromone-producing adoption glands located under the abdominal trichomes. Inside the nest, it lives as a socially integrated kleptoparasite, being fed by the host through trophallaxis. The beetle larvae are also fed through trophallaxis, but in addition to this also predate on ant brood. It is assumed that all *L. strumosus*-group species are similar to *L. strumosus* in the relationship to their respective *Formica* hosts. See Wasmann (1915) and Hölldobler et al. (2018) for detailed accounts of *L. strumosus*’ myrmecophilous habits.

##### Notes.

The larva is unknown but can safely be assumed to be similar to the well-known larva of *L. strumosus*, as all known larvae of both *Lomechusoides* and *Lomechusa* are generally very similar (Wasmann 1915).

##### Detailed comparison.

*Lomechusoides umbrosus* sp. nov. belongs to the *L. strumosus* complex within the *L. strumosus* species group sensu Jászay et al. (2023).

Although *L. umbrosus* sp. nov. has until now been confused with *L. strumosus*, it does not resemble it particularly much in comparison to other *Lomechusoides* species. *Lomechusoides umbrosus* sp. nov. shares more characteristics with the East Siberian *L. zerchei* and *L. primoricus* than with the European *L. strumosus, L. folgaricus* Jászay, Hlaváč & Baňař, 2023 and *L. wellenii*. These two East Siberian species, with *L. sibiricus* and *L. zeyai*, are the only other described members of the *strumosus* complex with a visible sharp edge on the posterior curved part of the pronotal lateral margins in lateral view. *Lomechusoides zeyai* is easily differentiated by its unique, strongly concave pronotal lateral margins and acute hind corners, a very different spermatheca, and smaller size (~5 mm). *Lomechusoides sibiricus* is easily differentiated by its acute hind corners of the pronotum, slightly concave lateral margins of the pronotum, and longer antennomeres (Jászay et al. 2023). However, *L. zerchei* and *L. primoricus* are more similar to *L. umbrosus* sp. nov.

*Lomechusoides zerchei* (Fig. 3D) is the only described member of the *L. strumosus* complex besides *L. umbrosus* sp. nov. where the pronotum is widest well in front of the posterior corners and narrows significantly posteriorly. It is easily differentiated from *L. umbrosus* sp. nov. by its distinctly trapezoidal pronotum shape, with the lateral margins being diverging and straight in the part anterior of the widest point, by having a much lighter reddish-brown head, lacking lateral lines on the metaventral process, and the transverse carinae on anterior parts of tergites II–V being disconnected in their entire length. The host of the examined *L. zerchei* specimens is a species in the *F. rufa* group.

*Lomechusoides primoricus* (Fig. 3C) is differentiated from *L. umbrosus* sp. nov. by having a dark brown head. Furthermore, it is differentiated by having 90-degree posterior corners of the pronotum, and widest point of the pronotum being at the posterior corners. The pronotal lateral margins of *L. primoricus* are much more parallel (sometimes slightly concave posteriorly) and less rounded and convex than in *L. umbrosus* sp. nov., making the pronotum appear much more square in *L. primoricus*. The length of the sharp edge on the lateral pronotal margin is also much longer in *L. primoricus*, where it covers the posterior third of the lateral margin. In *L. umbrosus* sp. nov. it is shorter and usually constrained to the curved part near the posterior corners. The host of the examined *L. primoricus* specimens is *F. sanguinea*.

*Lomechusoides zerchei, L. primoricus*, *L. sibiricus*, and *L. zeyai* are only known from regions east of Lake Baikal (Jászay et al. 2023), and all are generally lighter in colouration than the very dark *L. umbrosus* sp. nov.

##### European species.

In Europe, described members of the *L. strumosus* complex consist of *L. strumosus* (much of Europe), *L. wellenii* (Fennoscandia), *L. folgaricus* (The Alps), *L. rossii* Jászay, Hlaváč & Baňař, 2023 (Abruzzi), *L. siculus* Fiori, 1914 (Sicily), *L. teres* (Caucasus), *L. dudkorum* Jászay, Hlaváč & Baňař, 2023 (Urals and eastwards) and *L. richteri* Jászay, Hlaváč & Baňař, 2023 (Saratovsk, Volga). None of these species share pronotum shape with *L. umbrosus* sp. nov., and few overlap in range (Jászay et al. 2023). The Italian *L. folgaricus* and *L. rossii* share with *L. umbrosus* sp. nov. a glossy pronotal disc with very limited microsculpture but have either very weakly defined tubercles or entirely lack them. In *L. umbrosus* sp. nov., they are very well defined.

##### Northern European species.

**Figure 7.**
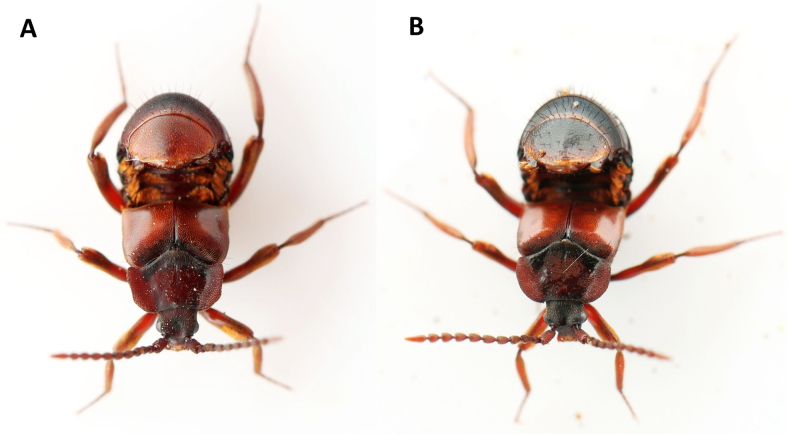
Typical resting posture of *Lomechusoides*. **A**. *Lomechusoides strumosus*. With *Formica sanguinea*, Nørlund Plantage, Denmark, 24 March 2025; **B**. *Lomechusoides umbrosus* sp. nov. (holotype). With *F. polyctena*, Hammarskog, Sweden, 20 September 2023. The two species are easily identified from photos of live animals by the stark difference in pronotum shape and glossiness, head colour, and abdomen colour.

In the Nordic Countries, *L. strumosus* (Figs 2A, 2C, 2E, 2F, 2I, 2K, 7A) and *L. wellenii* (Fig. 3B) of the *strumosus* complex, and *L. inflatus* (Fig. 3A) of the *inflatus* complex are the only other known representatives of the genus. *Lomechusoides umbrosus* sp. nov. is a large species, equal in size to *L. strumosus*, whereas the other known species *L. wellenii* and *L. inflatus* are slightly smaller to much smaller, respectively. In general, *L. umbrosus* sp. nov. has a much darker appearance than other Nordic *Lomechusoides* species, with both tergites and sternites being predominantly black, each bearing a thin brown stripe at the posterior margins. This is very apparent in life, when the abdomen is held in an upright position when the beetle is at rest. The jet black, glossy sternites of *L. umbrosus* sp. nov. (Fig. 7B) gives it a very different appearance than *L. strumosus*, who has a conspicuously vibrant deep red abdominal apex (Fig. 7A). This colour difference matches the general colour difference between their specific host ants, with *F. sanguinea* being more brightly red than the darker *F. rufa* group. On museum specimens the colour difference is often less clear than on living specimens, and colour characteristics should always be used together with morphological characteristics.

Among Nordic members of the genus, the spermatheca of *L. umbrosus* sp. nov. is unique in its apical and basal parts being approximately equal in length (basal part 0.88–1.08 × the length of apical part) (Fig. 4F), where the basal part is significantly longer than the apical part in *L. strumosus* (Fig. 4E), and significantly shorter in *L. wellenii* and *L. inflatus*. The spermatheca is usually also darker than in *L. strumosus*, which is especially apparent in the basal part.

##### Comparative material examined.

***Lomechusoides strumosus*: Austria** • 1♀; Hainburger Berge; 48.13, 16.93; leg. Scheerpeltz; ZMUH • 3♂♂, 5♀♀; Linz; 48.29, 14.31; 29.III.1913; leg. J. Kloiber; NHMW • 2♂♂, 2♀♀; Marzer Kogel, n. Marz-Rohrbach; 47.72, 16.45; I.IV.1926, leg. Scheerpeltz; NHMW [Pinned host: *Formica sanguinea*] • 5♂♂, 2♀♀; n. Graz; 47.07, 15.44; leg. Scheerpeltz; NHMW • 4♂♂, 6♀♀; Rekawinkel n. Vienna; 48.18, 16.03; leg. Scheerpeltz; NHMW [Pinned host: *Formica sanguinea*]. **Bosnia and Herzegovina** • 1♀; Nevesinje; 43.26, 18.11; leg. Schuster; NHMW. **Czechia** • 5♂♂, 4♀♀; Hannsdorf [Hanušovice]; 50.07, 16.93; leg. V. Zoufal; NHMW • 2♀♀; Jíloviště; 49.93, 14.34; 24.IV.1910; leg. K. Klenka; SMNS • 2♂♂, 5♀♀; Písecké Hory; 49.28, 14.23; 1917; leg. Scheerpeltz; NHMW [Pinned host: *Formica sanguinea*] • 1♀, 1♂; Pollauer Berge [Vrchy Pálavské]; 48.86, 16.64; 18.–19.IV.1924; leg. Scheerpeltz; NHMW [Pinned host: *Formica sanguinea*] • 1♂; Příbram; 49.67, 13.99; leg. J. Roubal; NHMW • 1 ex.; Tabor; 49.41, 14.68; leg. Luse; NHMW • 4 ex.; Zvole n. Prague; 49.93, 14.42; leg. Skalitzky; NHMW. **Denmark** • 2♂♂, 1♀; Feldborg Nørreskov; 56.36, 8.94; 22.IV.2025; leg. H. Kjærby; w. *Formica sanguinea*; cKjær • 1♀; Gludsted Plantage; 56.08, 9.36; 8.V.2024; leg. H. Kjærby; w. *Formica sanguinea*; cKjær • 2♂♂, 2♀♀; Hønning Plantage; 55.18, 8.95; 11.V.2025; leg. H. Kjærby; w. *Formica sanguinea*; cKjær • 1♀; Høstemark Skov; 56.93, 10.22; 17.VIII.2020; leg. H. Kjærby; w. *Formica sanguinea*; cKjær • 2♂♂, 4♀♀; Kompedal Plantage; 56.23, 9.26; 9.IV.2025; leg. H. Kjærby; w. *Formica sanguinea*; cKjær • 1♀; Melby Overdrev; 56.01, 11.99; 27.V.2025; leg. H. Kjærby; w. *Formica sanguinea*; cMart • 1♂; Nørlund Plantage; 56.05, 9.21; 24.III.2025; leg. H. Kjærby; w. *Formica sanguinea*; cKjær • 2♂♂; Nørlund Plantage; 56.05, 9.21; 3.IV.2025; leg. H. Kjærby; w. *Formica sanguinea*; cKjær • 1♂; Store Hjøllund Plantage; 56.05, 9.37; 17.VIII.2021; leg. H. Kjærby; w. *Formica sanguinea*; cKjær • 1♂, 1♀; Store Hjøllund Plantage; 56.05, 9.37; 18.IV.2024; leg. H. Kjærby; w. *Formica sanguinea*; cKjær • 1♀; Store Hjøllund Plantage, 56.05, 9.37, 21.III.2024, leg. H. Kjærby, w. *Formica sanguinea*; cKjær • 3♀♀; Store Hjøllund Plantage; 56.05, 9.37; 7.IV.2024; leg. H. Kjærby; *Formica sanguinea*; cKjær • 1♀; Store Hjøllund Plantage; 56.05, 9.37; 11.VI.2024; leg. H. Kjærby; *Formica sanguinea*; cKjær • 1♀; Tingskov; 57.08, 9.42; 22.IV.2023; leg. H. Kjærby; *Formica sanguinea*; cKjær • 1♂, 6♀♀; Tisvilde; 56.05, 12.06; 31.V.1908; leg. Rye; NHMA. **Estonia** • 1♀ Kõrkküla; 58.15, 22.37; 25.IV.–6.VI.2023; forest cut area window trap on aspen stand; TAMZ • 1♂; Aegviidu Pillapalu; 59.28, 25.61; 25.V.1994; TAMZ. **Finland** • 1♂; Helsinge [Helsinki]; 60.23, 24.99; leg. Karvonen; MZLU • 1♂; Helsingfors; 60.23, 24.99; leg. O. Wellenius; MZLU • 1♀; Joutseno; 61.12, 28.51; leg. E. Thuneberg; MZLU • 1♂, 3♀♀; Nylandia, Helsinki; 60.23, 24.99; 24.IV.1919; leg. Karvonen; ZMUO • 1♀; Pirkkala; 61.46, 23.64; leg. Grönblom; MZLU • 1♂, 2♀♀; Regio aboënsis, Raisio; 60.48, 22.16; 1940’s; leg. R. Linnavuori. ZMUO • 1♂; Regio aboënsis, Rymättylä; 60.37, 21.94; 1940’s; leg. R. Linnavuori. ZMUO • 1♂; Tampere; 61.49, 23.76; leg. Soyrinki; MZLU • 1♂, 1♀; Vehkalaliti; 60.57, 27.14; 2.VI.1962; leg. L. Tiensuu; MZLU. **France** • 1♀; Cote d’Or; 47.50, 4.64; leg. Rouget; SMNS • 1♀; Hautes Pyrénées; 42.99, 0.14; leg. Pandellé; NHMW. **Germany** • 4♂♂, 4♀♀; Asselfingen; 48.53, 10.19; 13.V.1952; SMNS • 1♀; Düsseldorf; 51.22, 6.78; leg. Dr. Friis; NHMW • 1♀; Eifel, 2 km ENE Schönecken; 50.17, 6.49; 7.IX.1991; leg. Schulz; Clearing in Pine Forest; NHMW • 1♀; Hannover, Helstorf Reiterheide; 52.58, 9.61; 30.V.1991; leg. V. Assing; NHMW • 1♂, 1♀; Kyffhauser; 51.11, 11.08; 2.V.1941; leg. Folwaczny; SMNS • Peutenhausen; 48.52, 11.23; 23.III.1974; leg. R Papperitz; SMNS • 2♂♂; Taubertal; 49.69, 9.63; 20.IV.1981; leg. R. Bickel; SMNS. **Greece** • 1♂; Parnassus, 38.55, 22.66; NHMW [Pinned host: *Formica sanguinea*]. **Italy** • 1♀; Val di Laux; 45.04, 7.03; 10.VI.1908; leg. Pinker; NHMW [Pinned host: *Formica sanguinea*] • 1♀; Valle di Saviore; 46.07, 10.43; VII.1855; leg. Cadamuro; NHMW. **Netherlands** • 1♂, 1♀; Exaten; 52.18, 6.41; V.1890; leg. Wasmann; NHMW [Pinned host: *Formica sanguinea*]. **Norway** • 1♀; Akershus, Vestby, Kroken; 59.56, 10.67; 9.V.1996; leg. S. Ligaard; cØde • 1♀; Buskerud, Kongsberg; 59.66, 9.65; 22.IV.1990; leg. B. Sagvolden; cØde• 1♀; Oppland, Nord fron Stordalsberge; 61.58, 9.81; 10.VII.1988; leg. O. Hanssen; In nest of *Formica sanguinea* at the root of fallen birch; cHans • 1♂; Oppland, Nord fron Stordalsberge; 61.61, 9.83; 4.VI.–3.IV.1998; leg. F. Ødegaard; pitfall trap with *Formica sanguinea*; cØde • 1♂; Oslo, Sogn; 59.95, 10.74; 15.V.1961; leg. J. Andersen; UiT. **Russia** • 1♀; Demidov distr., near Nikitenki; 55.49, 31.78; 22.–26.VII.2015; leg. O. Semionenkov; near a dead birch with *Formica sanguinea*; cSem • 1♀; Demidov distr., near Poboistche, SW part of “Kolpitski Mokh”; 55.46, 31.63; 1.–5.VII.2017; leg. O. Semionenkov; in nest of *Formica uralensis*, oligotrophic moor; cSem • 1♀; Ulyanovo Distr., “Kaluzhskiye Zaseki” Nature Reserve, Novaya Dyeryevnya; 53.59, 35.80; 10.–20.VI.2009; leg. S. Alekseev; window trap on a willow; cSem • 1♀; Vladimir area, Meshchera National Park, near Tasino; 55.52, 40.19; 13.VI.2008; leg. V.B. Semenov; cSem. **Slovakia** • 3♂♂, 4♀♀; Nyitra County; 48.31, 18.08; leg. H. Raffenberg; NHMW. **Sweden** • 1♀; Dalarna, St. Kopparberg; 60.61, 15.63; leg. Klefbeck; MZLU • 2♀♀; Halland, Släp; 57.51, 11.98; leg. Agren; MZLU • 2♂♂, 1♀; Skåne, Hässleholm; 56.16, 13.77; 7.V.1977; leg. F. Olsson; MZLU • 1♀; Skåne, Klostersågen; 55.64, 13.59: 15.V.1982; leg. H. Andersson; MZLU • 1♀, 1♂; Skåne, Vankiva; 56.19, 13.74; 9.V.1961; leg. F. Olsson; MZLU • 1♀, 1♂; Småland, Kalmar; 56.67, 16.32; 1.V.1980; leg. B. Anderson; MZLU • 1♀; Västra Götaland, Kråkhult; 57.71, 13.09; 29.VIII.1959; leg. H.-E. Wanntorp; cWann.

***Lomechusoides wellenii*: Finland** • 1♀; Helsinge [Helsinki]; 60.23, 24.99; 30.IV.1940; leg. O. Wellenius; w. *Formica uralensis*; NHMD • 1♀; Helsinge [Helsinki]; 30.IV.1940; leg. O. Wellenius; w. *Formica uralensis*; ZMUO. **Sweden** • 1♂ Dalarna, Norr Oradtjärnberg; 60.95, 14.18; 2006; leg. H.-E. Wanntorp; det. H. Kjærby; cWann • 1♀; unknown locality, leg. Gyllenhall; det. H. Kjærby; NHMD.

***Lomechusoides inflatus*: Norway** • 2♀♀; Tana, Torhop; 70.48, 27.98; 29.V.2024; leg. A. Staverløkk; w. *Formica gagatoides*; cKjær.

***Lomechusoides folgaricus*: Italy** • 3♂♂; Folgaria; 45.91, 11.18; leg. Winkler; NHMW • 1♂; Serraia; 46.13, 11.25; NHMW • 1♀; Colle St. Lucia; 46.45, 12.01; leg. Kaiser; NHMW.

***Lomechusoides teres*: Georgia** • 3♂♂; Caucasus, Lomis Mts.; 41.87 43.25; 2100 m; leg. König, NHMW [Pinned host: *Formica sanguinea*]. **Turkey** • 1♂; Trabzon, Doğu Karadeniz Dağları, Maçka, Fırınoba plateau environs; 2200 m; 40.6285, 39.6866, 14.V.2025; leg. A. Solodovnikov; alpine zone; under stones; NHMD.

***Lomechusoides primoricus*: Paratypes Russia** • 1♂ 1♀; Primorskij kray [=Primorsky Kray], Sikhote-Alin, Meteorol. St., 28 km SE Chuguyevka; 43.59, 134.08; 900 m; 1.VI.1993; leg. Zerche; SDEI [Pinned host: *Formica sanguinea*].

***Lomechusoides zerchei*: Paratypes Russia** • 2♂♂ 2♀♀; Primorsky kray, Anisimovka (=Kangaus), Krinichnaya Mt., 70 km E Vladivostok; 43.07, 132.41; 500 m; 8.VI.1993; leg. L. Zerche; SDEI [Pinned host: *Formica rufa* group].

***Lomechusoides dudkorum*: Holotype Russia** • ♂; 6 км к югу от г. Тюмени [6 km k yugu ot g. Tyumeni (=6 km to the south from town Tyumen)]; 57.07, 65.55; 3.IV.1995; leg. *A. et*. R. Dudko; NHMD [Pinned host: *Formica sanguinea*] **Paratypes** • 2♀♀; 6 км к югу от г. Тюмени [=6 km to the south from town Tyumen]; 57.07, 65.55; 3.IV.1995; leg. *A. et*. R. Dudko; NHMD.

***Lomechusoides mongolicus*: Russia** • 1♀; Buryatia Rep., Selenga R. 8 km N Tarbagataj; 51.68, 108.41; 28.V..2002; leg. A. Anistschenko; NHMD • 1♀; Даурия [Chita area], оз. Барун-Торей, протока Утыча на [Lake Barun-Torey, Utych Strait]; 50.02, 115.39; 1.VI.2000; O. Корсун [O. Korsun] leg.; NHMD.

***Lomechusoides fallax*: Paratype Russia** • 1♀; Primorsky kray, Anisimovka (=Kangaus), Krinichnaya Mt., 70 km E Vladivostok; 43.07, 132.41; 500 m; 8.VI.1993; leg. L. Zerche; SDEI [Pinned host: *Formica rufa* group].

### Key to the *Lomechusoides strumosus* species group of the world

To be inserted into the key of Jászay et al. (2023: 543) by replacing the current point 13 to create a 13a and 13b.

**Table:** 

13a	Pronotal lateral margins broadly rounded. Pronotum widest at posterior ¼–⅓. Posteriorly slightly convergent, making the posterior corners obtuse. This gives the lateral margins as a whole a semicircular and convex shape. Southern-Central Fennoscandia, Eastern Europe	***Lomechusoides umbrosus* sp. nov**.
–	Pronotal lateral margins subparallel posteriorly, pronotum widest at posterior corners or immediately anterior thereof	**13b**
13b	Surface of lateral impressions on pronotum evenly microsculptured, disc of pronotum lacking microsculpture, shiny; lateral sides of pronotum straight or slightly concave; anterior third of lateral margins of pronotum thickened in lateral view, equally thin posteriad, with visible sharp edge in posterior third; elytra with rasp-like punctures; Russia: Far East	***Lomechusoides primoricus* Jászay et al., 2023**
–	Surface of lateral impressions on pronotum in basal part unevenly microsculptured, disc of pronotum with uneven microsculpture, slightly shiny; lateral sides of pronotum slightly convex; anterior third of lateral margins of pronotum thickened in lateral view, margins thickest at middle, becoming thinner toward anterior and posterior angles, lacking visible sharp edge; elytra lacking rasp-like punctures; Finland and Sweden	***Lomechusoides wellenii* (Palm, 1949)**

### Simplified key to the *Lomechusoides* species of the Nordic Countries

**Table:** 

1	Lateral sides of pronotum lacking macrosetae. Anterior part of elytra with 2–4 macrosetae. Small species (~4.5–5 mm). Distribution: Northern (arctic and sub-arctic) Fennoscandia. Host ant: Formica (Serviformica) gagatoides	***Lomechusoides inflatus* Zetterstedt, 1828**
–	Lateral sides of pronotum with 4–7 macrosetae. Anterior part of elytra with 6–9 macrosetae. Larger species (~5–7 mm). *Lomechusoides strumosus* complex	**2**
2	Pronotum widest at ¼–⅓ of the marginal length from posterior corners. Lateral margins converging behind widest point. Pronotal lateral margins broadly rounded in their entire length. Pronotal lateral margin with a sharp edge near posterior corners. Transverse carinae on anterior part of visible tergites II–V connected in the middle by one or more broad septa. Frontal median impression on head glossy, with microsculpture uneven and barely visible, much weaker than rest of head. Head black. Large (~6–7 mm). Basal part of spermatheca roughly equal in length to apical part. Distribution: Southern-Central Fennoscandia, Eastern Europe. Host ant: Formica (Formica) rufa group (especially *F. polyctena*)	***Lomechusoides umbrosus* sp. nov**.
–	Pronotum widest at posterior corners or immediately anterior thereof. Lateral margins subparallel posteriorly. Pronotal lateral margins with blunt edge. Transverse carinae on anterior part of visible tergite II to V disconnected in their entire length. Frontal median impression on head dull, with microsculpture slightly weaker than rest of head. Head black or brown. Large (~6–7 mm) or smaller (~5–6 mm)	**3**
3	Antennomeres very slender. Pronotal lateral margins subparallel posteriorly, then relatively smoothly narrowed frontally. Pronotal disc weakly and unevenly microsculptured, rather glossy. Eyes protuberant. Head black. Smaller (~5–6 mm). Basal part of spermatheca much shorter than apical part. Distribution: Central-Northern Fennoscandia (Sweden, Finland). Host ant: *Formica uralensis, F. (Formica) rufa group*	***Lomechusoides wellenii* (Palm, 1949)**
–	Antennomeres very robust. Pronotal lateral margins subparallel posteriorly, then suddenly narrowed frontally, creating an angled bend. Pronotal disc with light microsculpture all over, dull. Eyes flat or slightly protuberant. Head brown. Larger (~6–7 mm). Basal part of spermatheca longer than apical part. Distribution: Europe, entirety of the Nordics. Host ant: Formica (Raptiformica) sanguinea	***Lomechusoides strumosus* (Fabricius, 1792)**

## Discussion

### Integrative evidence for species delimitation under low divergence

Despite the relatively shallow COI barcode divergence we observe between *Lomechusoides umbrosus* sp. nov. and its closest congeners (0.7–1.4%), our results align with a growing body of work showing that low interspecific COI distances do not preclude clear species limits when stable, diagnostic morphology is present. In *Quedius* s. str., for example, very little COI divergence separates the unambiguously distinct species *Q. hispanicus* Bernhauer, 1898 and *Q. pallipes* Lucas, 1849, underscoring that morphology can remain a decisive line of evidence even where barcodes are conservative (Hansen et al. 2022). A similar situation exists for the species pair *Philonthus varians* (Paykull, 1789) and *P. pseudovarians* A. Strand, 1941 (AKH unpublished data). Likewise, integrative analyses have elevated *Philonthus sideropterus* Kolenati, 1846 from a colour form of *P. splendens* (Fabricius, 1793) to full species based on consistent morphological differences supported by barcodes and distributional data, despite modest mitochondrial distances (4.3%) (Geisler et al. 2024). Conversely, barcodes can reveal conspecificity when strikingly dissimilar morphotypes do not represent separate species, as has been shown for the high-altitude morph of *Lobrathium multipunctum* (Gravenhorst, 1802), cautioning against over-reliance on external variation alone (Hansen and Jenkins Shaw 2023). Importantly, the genetic differences between our newly proposed *Lomechusoides* species are comparable to those separating already accepted congeners, and the species retains sets of stable, non-overlapping morphological characters. Additionally, this decision is further supported by strong bionomic data on host choice. All these together provide congruent evidence for the species status even in low COI divergence scenarios and illustrate the power of an integrative approach when evaluating taxonomic decisions.

### Distribution and expected range

The currently collected specimens of *L. umbrosus* sp. nov. indicate a broad potential distribution in Northern and Eastern Europe, at least from Scania in the west to the Urals in the east, and from Uppland in the north to Bialowieza in the south. Its range can reasonably be expected to include all of southern and central Sweden between the site in Scania and the Uppland distribution, as well as all of southern Finland. Perhaps it occurs further north in Fennoscandia as well, with the northern distribution limit depending on whether *F. aquilonia* Yarrow, 1955 or *F. lugubris* Zetterstedt, 1838 can serve as hosts or not. The Nordic distribution could also reach further west to Denmark and Norway. It is also very likely to occur in all the Baltic States, all of Belarus, Northern Ukraine, much of European Russia, and possibly the Carpathians. The host ant is common in much of Europe, and it is not unlikely that the distribution of *L. umbrosus* sp. nov. could reach as far west as Germany. However, the lack of *L. umbrosus* sp. nov. among the examined Central European museum collections of *L. strumosus* indicates that the new species is likely missing in much of Central Europe, or rare if present. Lomechusoides umbrosus sp. nov. could occur further east in Siberia as well, but probably not beyond the Reinig Line faunal divide (De Lattin 1967).

### Host associations and ecology

In every case where the host ant was documented for collected *L. umbrosus* sp. nov. specimens, it was *F. polyctena*. For the Scanian Vittsjö series from 1980 (MZLU collection), *F. rufa* is noted as host. Whether this is to be understood as *F. rufa* sensu lato or sensu stricto is unknown, and no host ants were collected alongside the series. Both *F. rufa* and *F. polyctena* are found in the forests around Vittsjö. Three of the authors (HSK, SBBC, AKH) searched many nests of both species in April 2025 without rediscovering *L. umbrosus* sp. nov. in the area.

Even though *F. polyctena* is currently the only documented host, it is not impossible that other members of the *F. rufa* group (probably *F. rufa* and/or *F. aquilonia*, possibly *F. lugubris* and/or *F. pratensis*) may serve as hosts for *L. umbrosus* sp. nov. Hybridisation frequently occurs in this group. Especially *F. rufa*, *F. polyctena*, and *F. aquilonia* hybridise willingly, often making identification difficult and locally blurring species delimitation. In Finland, nearly all *F. polyctena* seem to have admixture from *F. aquilonia* (Pamilo and Kulmuni 2022). *Formica polyctena* and *F. aquilonia* are both polygynous and polydomous, and thus similar in ecology, whilst the usually monogynous and monodomous *F. rufa* is the closest phylogenetic relative of *F. polyctena* (Pamilo and Kulmuni 2022).

### Host specificity and ecological differentiation

The host of *Lomechusoides strumosus*, F. (Raptiformica) sanguinea, belongs to a separate subgenus than the host of *L. umbrosus* sp. nov., F. (Formica) polyctena, and have distinct differences in behaviour, nest construction, and habitat choice. Therefore, it is very implausible that *L. umbrosus* sp. nov. and *L. strumosus* are found in the same ant nests, despite overlapping throughout all of the former’s known distribution. The two beetles also differ in their physical appearance, where they curiously seem to mirror their respective host in colour, the dark *L. umbrosus* sp. nov. resembling the darker *F. polyctena* and the lighter *L. strumosus* resembling the paler *F. sanguinea*.

The consistent host association observed for *L. strumosus* and *L. umbrosus* sp. nov. suggests that most *Lomechusoides* species are likely host specific, occurring with only one or a few closely related *Formica* species. A single *Formica* species may however be host to several species of *Lomechusoides* throughout its range, as seems to be the case for at least *F. sanguinea*, which has a massive trans-Eurasian distribution. This ant species hosts, in addition to *L. strumosus*, at least *L. dudkorum*, *L. teres*, and *L. primoricus*.

In the case of *L. strumosus*, several newer sources cite *F. rufa, F. polyctena, F. pratensis, F. fusca*, *F. rufibarbis*, and even *Myrmica rubra* (Linnaeus, 1758) and *M. scabrinodis* Nylander, 1846 as regular hosts, often without highlighting *F. sanguinea* (Päivinen et al. 2002; Hlaváč et al. 2011; Harrison and Albena 2014; Jászay et al. 2023). This must be considered incorrect; *Formica sanguinea* has been well documented to be the specific host of *L. strumosus* since the 1800’s, especially with the very extensive field observations of Wasmann (1915) made in Dutch Limburg over several decades. If *L. strumosus* truly had as many regular host species as sometimes cited in newer literature, it would be expected to breed in nests of more than one *Formica* species on sites where several occur. However, similarly to Wasmann (1915), HSK’s extensive field observations from several sites in Jutland exclusively show *L. strumosus* in the nests of *F. sanguinea*, and despite repeated searching, not in any neighbouring nests of *F. rufa, F. polyctena, F. pratensis*, or *F. truncorum*, even when these species nest less than 10 meters from infested *F. sanguinea* nests. A similar pattern has been observed by HSK with *L. inflatus* on a site in Northern Sweden. *Lomechusoides inflatus* only occurred with F. (Serviformica) gagatoides Ruzsky, 1904 on the site, where nests of the closely related F. (Serviformica) lemani Bondroit, 1917 were much more numerous, and the two ant species often nested in very close proximity to each other (HSK unpublished results). Likewise, *F. sanguinea* is the sole represented host ant with the examined museum specimens that could be confidently assigned to *L. strumosus*. One exception is a single specimen from Smolensk collected with *F. uralensis*. We identify the specimen as *L. strumosus*, and its presence in a nest of *F. uralensis* could be incidental, as *L. strumosus* has also been collected nearby with *F. sanguinea*. Rare, isolated cases of individual *L. strumosus* in nests of *F. rufa* and *F. pratensis* are reported by Wasmann (1915). These are undoubtedly incidental, as *L. strumosus*-infested *F. sanguinea* nests were always close by, from which he argues the beetles surely originated (Wasmann 1915). In any case, the massive overrepresentation of *F. sanguinea* as host must be interpreted as host specificity.

Unusual host records should be treated with greater attention in future studies. Reports of “*L. strumosus*” living with *F. polyctena* have existed for decades and could have alerted entomologists to the existence of *L. umbrosus* sp. nov. long ago. While differences in host ants alone cannot define species boundaries, they often signal ecological divergence worth investigating, and as was the case in this study it can lead to the discovery and recognition of overlooked species.

Ecological knowledge on most *Lomechusoides* species is unfortunately severely lacking. It is possible that some of the unusual hosts attributed to *L. strumosus* are not accidental occurrences, but due to these ants instead being the hosts of recently described *Lomechusoides* species, such as those by Jászay et al. (2023). Many pinned specimens include associated host ants, but no systematic examination of the hosts has ever been conducted. A complete catalogue of the host relationships of *Lomechusoides* would be valuable and we encourage myrmecologists to start this work.

## Conclusions

In recent years, several taxonomic works have revealed considerable overlooked diversity within *Lomechusoides* across Eurasia. However, ecological knowledge on both newly described and known species is still lacking, and information regarding host relationships has been increasingly muddied. The strict association of *L. strumosus* with *Formica sanguinea* is historically well-documented and described extensively in the literature (Wasmann 1915) but has not been corroborated by several newer sources. Our examination of *Lomechusoides* specimens associated with *F. polyctena* formerly assigned to *L. strumosus* revealed them to represent a hitherto unrecognised species, *L. umbrosus* sp. nov. Thus, our findings support Wasmann’s characterisation of host specificity in *L. strumosus*. Based on the observed patterns, we suggest that *Lomechusoides* species are generally relatively host specific.

For most other *Lomechusoides* species, there is unfortunately either a lack of ecological data, or conflicting reports on host ant species. By clarifying species boundaries and documenting host specificity, this study provides a foundation for future research on the ecological and evolutionary dynamics of myrmecophily in the genus. Together with the extensive revision by Jászay et al. (2023) it paves the way for renewed integrative studies on host use, behavioural adaptation, and the coevolutionary history of this remarkable genus of ant-associated beetles.

## Supplementary Material

XML Treatment for Lomechusoides umbrosus

## Additional information

### Use of AI

No use of AI was reported.

### Author ORCIDs

Hjalte Sebastian Kjærby https://orcid.org/0009-0003-6436-185X

Sean Birk Bek Craig https://orcid.org/0009-0003-9142-7762

Philip Francis Thomsen https://orcid.org/0000-0002-9867-4366

Aslak Kappel Hansen https://orcid.org/0000-0003-2089-7233

### Data availability

All of the data that support the findings of this study are available in the main text or Supplementary Information.

## Supplementary materials

Supplementary material 1 Fasta-file containing barcodes used in the phylogeny Data type fasta https://binary.pensoft.net/file/1573702This dataset is made available under the Open Database License (http://opendatacommons.org/licenses/odbl/1.0/). The Open Database License (ODbL) is a license agreement intended to allow users to freely share, modify, and use this Dataset while maintaining this same freedom for others, provided that the original source and author(s) are credited. Hjalte Sebastian Kjærby, Sean Birk Bek Craig, Philip Francis Thomsen, Aslak Kappel Hansen

Supplementary material 2 Overview of examined material Data type xlsx https://binary.pensoft.net/file/1573703This dataset is made available under the Open Database License (http://opendatacommons.org/licenses/odbl/1.0/). The Open Database License (ODbL) is a license agreement intended to allow users to freely share, modify, and use this Dataset while maintaining this same freedom for others, provided that the original source and author(s) are credited. Hjalte Sebastian Kjærby, Sean Birk Bek Craig, Philip Francis Thomsen, Aslak Kappel Hansen

Supplementary material 3 Metadata for specimens with barcodes Data type xlsx https://binary.pensoft.net/file/1573704This dataset is made available under the Open Database License (http://opendatacommons.org/licenses/odbl/1.0/). The Open Database License (ODbL) is a license agreement intended to allow users to freely share, modify, and use this Dataset while maintaining this same freedom for others, provided that the original source and author(s) are credited. Hjalte Sebastian Kjærby, Sean Birk Bek Craig, Philip Francis Thomsen, Aslak Kappel Hansen
